# Mesoporous Bioactive Glass Nanoparticle-Reinforced Calcium Silicate Sealer for Reduced Microleakage and Enhanced Antibacterial Performance

**DOI:** 10.3390/jfb17070338

**Published:** 2026-07-13

**Authors:** Zun Zhang, Qianqian Zhang, Ying Sun, Baiyan Sui, Xin Liu

**Affiliations:** 1 Department of Stomatology, Shanghai East Hospital, Tongji University, Shanghai 200120, China; zz5609@easthospital.cn; 2 Department of Dental Materials, Shanghai Biomaterials Research & Testing Center, Shanghai Ninth People’s Hospital, Shanghai Jiao Tong University School of Medicine, College of Stomatology, Shanghai Jiao Tong University, Shanghai 200011, China; zhangqianqian@sh9hospital.org.cn (Q.Z.); sunying@sh9hospital.org.cn (Y.S.)

**Keywords:** root canal sealer, mesoporous bioactive glass nanoparticles, apical microleakage, dentin remineralization, antibacterial activity

## Abstract

Long-term success of root canal therapy depends not only on effective disinfection but also on durable sealing of the obturated canal system. However, currently available sealers still face persistent challenges in balancing handling, interfacial stability, bioactivity, and antibacterial performance. Here, we developed an injectable calcium silicate-based root canal sealer reinforced with mesoporous bioactive glass nanoparticles (MBGN) to improve sealing-related performance. The formulation integrated a hydration-active calcium silicate matrix with a mesoporous bioactive component while maintaining practical handling characteristics. MBGN incorporation enhanced dentin-associated mineralization, promoted intratubular crystal deposition, reduced apical microleakage, and decreased internal porosity after obturation. The 5% MBG formulation showed the most favorable sealing profile, reducing the dye penetration depth from 2.68 ± 0.41 mm in the 0% MBG group to 1.87 ± 0.32 mm, together with decreased open and closed pore parameters in the apical region. In parallel, the MBGN-reinforced sealer preserved acceptable cytocompatibility and exhibited stronger antibacterial activity against Streptococcus mutans than the reference formulations. The improved performance may be associated with effective initial adaptation and bioactive interfacial densification. Together, these findings suggest that MBGN incorporation may be a promising route for engineering more bioactive calcium silicate sealers with improved sealing stability and antibacterial function for endodontic applications.

## 1. Introduction

Apical periodontitis remains a common oral inflammatory disease and a major cause of tooth loss worldwide, imposing a substantial burden on oral function and quality of life [1]. Root canal treatment is currently the principal strategy for eliminating intracanal infection and preserving natural dentition [2]. However, the long-term success of endodontic therapy depends not only on effective microbial control, but also on the establishment and maintenance of a durable seal that prevents reinfection through leakage at the sealer–dentin interface [3,4]. In this regard, interfacial defects, void formation, material dissolution, and incomplete adaptation may create leakage pathways that permit bacterial recolonization and compromise treatment outcomes over time [3,4,5]. Therefore, the clinical challenge of root canal sealing is not merely to occupy the canal space, but to achieve long-term interfacial integrity and resistance to microleakage after obturation.

Root canal sealers are expected to fill anatomical irregularities, reduce gaps between gutta-percha and canal walls, and contribute to the biological stability of the obturated canal system. Conventional root canal sealers, including zinc oxide eugenol-based and epoxy resin-based sealers, have been widely used because of their acceptable handling or sealing performance [6,7,8]. However, the simultaneous achievement of practical handling, reliable antibacterial activity, bioactivity, and long-term interfacial sealing remains challenging for current root canal sealers. Among alkaline and bioactive materials, calcium hydroxide-based sealers or pastes, such as Vitapex, can create an alkaline antibacterial environment and have been used clinically because of their bioactivity and handling convenience [9,10]. Nevertheless, their sealing durability may be affected by dissolution, limited interfacial stability, and insufficient formation of a stable dentin-associated mineral interface [11,12]. Calcium silicate chemistry has provided an important foundation for bioactive endodontic materials, with mineral trioxide aggregate (MTA) being a representative early example [13]. MTA exhibits favorable sealing ability, biocompatibility, and bioactivity, but its broader use is limited by long setting time, handling inconvenience, potential discoloration, and relatively high cost [14]. More recently, premixed calcium silicate-based sealers, such as iRoot SP, have been developed to improve operability and clinical applicability. These sealers generally show favorable biocompatibility, alkaline pH, and apatite-forming ability; however, further improvement is still needed to optimize their sealing-related performance, particularly in terms of dimensional stability, interfacial adaptation, internal porosity, microleakage resistance, and durable antibacterial activity [8,15]. These limitations highlight the need for root canal sealers that combine practical handling with bioactive interfacial sealing capacity.

Bioactive glass has been widely investigated in dental and endodontic materials owing to its ion-releasing behavior, ability to induce hydroxyapatite formation, and intrinsic antibacterial activity in biological environments [16,17]. In calcium silicate-based root canal sealers, bioactive glass-related strategies have also been explored to further improve physicochemical and biological performance. For example, a previous study reported a premixed calcium silicate-based root canal sealer reinforced with bioactive glass nanoparticles and demonstrated improved biological properties and mineralization-related behavior [18]. These findings support the feasibility of using bioactive glass particles as functional additives in calcium silicate-based sealers. Building on this evidence, the present work focuses on translating bioactive glass-mediated mineralization into sealing-related functional outcomes, rather than only improving general bioactivity.

Compared with conventional bioactive glass particles, mesoporous bioactive glass (MBG) possesses a high specific surface area, accessible mesoporous channels, and enhanced ion-exchange capability, which collectively support accelerated apatite formation, sustained release of calcium- and silicate-related species, and local microenvironment modulation [19,20,21,22]. These established features make MBG nanoparticles (MBGN) particularly attractive for endodontic material design. From the perspective of root canal obturation, MBGN are expected to act not merely as passive inorganic fillers, but as a bioactive functional phase capable of promoting localized mineral deposition along dentinal tubules and at the sealer–dentin interface. Such ion-mediated interfacial mineralization may promote the occlusion of dentinal tubules and interfacial defects, thereby reducing potential leakage pathways and contributing to post-obturation interface densification [23,24].

Based on this rationale, we developed an injectable MBGN-reinforced calcium silicate sealer and specifically considered MBGN as a functional phase for interfacial sealing reinforcement. The sealer was evaluated in terms of handling-related properties, cytocompatibility, dentin-associated mineralization, internal porosity, sealer–dentin interfacial adaptation, apical microleakage, and antibacterial activity. This work was designed to connect the mineralization-promoting and ion-releasing properties of MBGN with improved interfacial sealing and antibacterial performance in an endodontic obturation system.

## 2. Materials and Methods

### 2.1. Materials

Tetraethyl orthosilicate (TEOS, ≥99.0%, Sigma-Aldrich, Hamburg, Germany), calcium nitrate tetrahydrate (Ca(NO_3_)_2_·4H_2_O, VWR International, Radnor, PA, USA), ethyl acetate (≥99.5%, Sigma-Aldrich, Hamburg, Germany), cetyltrimethylammonium bromide (CTAB, Merck, Darmstadt, Germany), and ammonium hydroxide (28 wt%, Merck, Darmstadt, Germany) were used for synthesis of MBGNs. Calcium carbonate (Aladdin, Shanghai, China), zirconium dioxide (Macklin, Shanghai, China), EDTA root canal lubricant (Langli Bio, Wuhan, China), and sodium hypochlorite irrigation solutions (2% and 5.25%, Langli Bio, Wuhan, China) were used for sealer preparation and root canal procedures. iRoot SP bioceramic root canal sealer (Innovative BioCeramix Inc., Vancouver, BC, Canada) and Vitapex root canal filling paste (Morita, Osaka, Japan) were used as clinical reference materials. Methylene blue solution (1%, Solarbio, Beijing, China), MTT (Thiazolyl Blue, VWR International, Radnor, PA, USA), L929 mouse fibroblasts (National Collection of Authenticated Cell Cultures, Shanghai, China), fetal bovine serum (FBS, Capricorn Scientific, Shanghai, China), MEM medium (Thermo Fisher Scientific Inc., Waltham, MA, USA), Brain Heart Infusion broth (BHI, Huankai Biology, Guangzhou, China), and Streptococcus mutans UA159 (Shanghai Institute of Biochemistry and Cell Biology, Chinese Academy of Sciences, Shanghai, China) were used for biological evaluation. Unless otherwise specified, all reagents were of analytical grade and used as received.

### 2.2. Synthesis of MBGNs and Preparation of Root Canal Sealers

MBGN were synthesized according to previously reported procedures with minor modifications [25]. Briefly, 5.6 g CTAB was dissolved in 264 mL deionized water obtained from a water purification system (PURELAB Option, ELGA LabWater, Lane End, UK) under continuous stirring for 30 min, followed by the addition of 80 mL ethyl acetate. After an additional 30 min, 56 mL aqueous ammonia (1 M) was added and stirred for 15 min. Subsequently, 28.8 mL TEOS and 18.24 g Ca(NO_3_)_2_·4H_2_O were sequentially introduced at 30 min intervals. The mixture was stirred for a further 4 h to form a colloidal suspension. The precipitate was collected by centrifugation using a refrigerated centrifuge (5430R, Eppendorf SE, Hamburg, Germany), washed with deionized water and ethanol, dried overnight at 60 °C in an electric oven (DRY-Line^®^, VWR, Radnor, PA, USA). Finally, the dried powder was calcined at 700 °C for 3 h at a heating rate of 2 °C/min in a furnace (P330, Nabertherm, Lilienthal, Germany) to obtain MBGNs.

The morphology and elemental composition of MBGN were examined using scanning electron microscopy (SEM, Tescan Mira 3 XH, Tescan, Brno, Czech Republic), transmission electron microscopy (TEM, JEOL 2100F, JEOL Ltd., Tokyo, Japan), and energy-dispersive spectroscopy (EDS). Fourier transform infrared spectroscopy (FTIR, Nicolet iS50, Thermo Scientific, Waltham, MA, USA) was performed using KBr pellets at a resolution of 4 cm^−1^, 64 scans, and a spectral range of 4000–400 cm^−1^.

The experimental sealers were composed of tricalcium silicate (C_3_S), dicalcium silicate (C_2_S), zirconium dioxide, calcium carbonate, and MBGN, with PEG 200 and Tween-80 as liquid carriers. Formulations containing 0, 1, 3, and 5 wt% MBGN were prepared and designated as 0% MBG, 1% MBG, 3% MBG, and 5% MBG, respectively. These MBGN-reinforced formulations were selected to represent low, intermediate, and relatively high incorporation levels, allowing evaluation of the effect of MBGN content on the sealer system while maintaining paste homogeneity and handling properties. The liquid carrier consisted of 95 wt% PEG 200 and 5 wt% Tween-80. The powder compositions of the experimental sealers are listed in Table 1, and the overall compositions of the tested materials are summarized in Table 2. iRoot SP and Vitapex were used as clinical reference sealers.

### 2.3. Flowability and Film Thickness Test

Flowability and film thickness tests were measured according to ISO 6876 [27]. For the flowability test, 0.05 mL freshly mixed sealer was placed at the center of a glass plate. After 180 s, a second glass plate (20 g) was placed on top, followed by a 100 g load. After 10 min, the maximum and minimum diameters of the compressed disc were measured. When the difference between the two diameters was less than 1 mm, the mean value was recorded. Three specimens were tested for each group.

For film thickness, the thickness of two superimposed glass plates was measured using a vernier caliper before testing. A defined amount of freshly mixed sealer was placed between the plates, followed by application of a vertical load of 150 N using a universal testing machine. After 10 min, the combined thickness was measured again, and the film thickness was calculated as the difference between the two measurements. Three specimens were tested per group.

### 2.4. Setting Time Test

Setting time was measured under conditions consistent with ISO 6876 [27]. Freshly mixed sealer was placed into high-density gypsum molds (10 mm in diameter and 1 mm in height) and stored at 37 °C under 100% relative humidity. A needle penetrometer with a 100 g mass and a 2 mm flat-ended tip was gently lowered vertically onto the specimen surface at predetermined time intervals. Initial and final setting times were recorded according to the disappearance of visible surface indentation under the applied load. Three specimens were tested per group.

### 2.5. Dentin Remineralization Assay

The collection and use of extracted human teeth were approved by the Ethics Committee of Shanghai Ninth People’s Hospital, Shanghai Jiao Tong University School of Medicine (approval no. SH9H-2025-T293-1). Extracted human teeth were cleaned by ultrasonic scaling to remove debris and residual soft tissue, disinfected in 5.25% sodium hypochlorite for 1 h, and stored in PBS containing 2% penicillin–streptomycin at 4 °C before use. One-millimeter-thick dentin discs were prepared below the enamel–dentin junction using a hard-tissue microtome. The discs were sequentially treated with 0.5% sodium hypochlorite for 5 min, rinsed with deionized water, disinfected with 70% ethanol for 20 min, and fully demineralized with 10% phosphoric acid for 12 h at 25 °C. The tested sealers were uniformly applied to the dentin surface, and the specimens were immersed in simulated body fluid (SBF, pH 7.4) at 37 °C for 21 days without refreshing the medium. After incubation, residual sealer was removed, and the specimens were air-dried. Surface and cross-sectional morphologies were observed using SEM-EDS (Tescan Mira 3 XH, Tescan, Brno, Czech Republic), and elemental analysis was performed to assess mineral deposition.

### 2.6. Root Canal Filling and Sealing Evaluation

#### 2.6.1. Root Canal Preparation and Obturation

Forty-eight intact premolars extracted for orthodontic reasons were collected. After removal of soft tissue remnants and ultrasonic cleaning, the teeth were disinfected in 5.25% sodium hypochlorite for 1 h. The crowns were removed at the cementoenamel junction, and the root length was standardized to 12 mm. Pulp tissue was removed using a barbed broach, and root canals were prepared with K-files. The working length was established at 0.5 mm short of the apical foramen. After instrumentation, canals were irrigated, rinsed with saline, and dried with paper points. The teeth were randomly assigned to four groups (*n* = 12): 0% MBG, 5% MBG, iRoot SP, and Vitapex. Root canals were obturated using the single-cone technique with matched gutta-percha cones coated with sealer. Each cone was inserted to working length with gentle vertical compaction to improve adaptation and minimize void formation. Excess gutta-percha was removed with a heated plugger at the canal orifice. The specimens were stored at 37 °C and 100% humidity for 7 days to allow sufficient hardening before further evaluation.

#### 2.6.2. Micro-CT Analysis

Micro-CT (nanoVoxel-2000, Sanying MotionControl, Tianjin, China) was used to evaluate filling quality. Scanning parameters were as follows: voltage 85 kV, current 100 μA, isotropic resolution 9.99 μm, 360° rotation, and 0.5° per frame. Three-dimensional reconstruction was performed using VoxelStudio Recon (2.5.1.25), and image analysis was conducted using CTvox (3.2) and Dragonfly (2022.2). The filled root canal space extending 0–9 mm from the apex was defined as the region of interest and further divided into apical (0–3 mm), middle (3–6 mm), and coronal (6–9 mm) thirds. Image segmentation was performed using grayscale thresholding based on the histogram distribution and visual inspection of representative slices to distinguish dentin, filling material, and voids. The same thresholding protocol was applied to all specimens to ensure consistency. Based on three-dimensional connectivity analysis, open pores were defined as voids or interfacial gaps connected to the canal boundary, whereas closed pores were defined as isolated voids completely enclosed within the filling material and without communication with the canal wall or external surface. The open and closed pore parameters were quantified in each root third. Six specimens from each group were used for micro-CT analysis.

#### 2.6.3. SEM Observation of the Sealer-Dentin Interface

After the 7-day hardening period, specimens were embedded in light-curing resin and sectioned longitudinally along the root axis. The sections were ultrasonically cleaned, vacuum-dried, sputter-coated with gold, and observed by SEM to evaluate interfacial adaptation and sealer penetration into dentinal tubules.

#### 2.6.4. Dye Penetration Test

Apical sealing ability was evaluated by dye penetration. After complete setting, the external root surfaces were coated with three layers of transparent nail varnish, leaving the apical 2 mm exposed. The specimens were mounted vertically with the root apex facing downward and immersed to a depth of 3 mm from the apex in 1% methylene blue solution. Samples were stored at 37 °C under 100% humidity for 7 days. After immersion, the roots were rinsed, embedded in light-cured resin, and sectioned longitudinally along the central axis. Dye penetration depth was measured under a stereomicroscope at 20× magnification.

### 2.7. Cytotoxicity Assay

Cytocompatibility was evaluated using the MTT assay. Extracts of the experimental sealers (0%, 1%, 3%, and 5% MBG) and iRoot SP were prepared in MEM supplemented with 10% FBS at a mass-to-volume ratio of 1:10 (1 g/10 mL) under sterile conditions at 37 °C for 24 h. L929 cells were seeded in 96-well plates at a density of 1 × 10^4^ cells per well and cultured for 24 h. Material extracts were diluted with MEM to final concentrations of 25%, 50%, 75%, and 100%, added to the cells at 100 μL per well, and incubated for a further 24 h. Subsequently, 50 μL MTT solution (1 mg/mL) was added to each well and incubated for 2 h at 37 °C. The medium was removed, and 100 μL isopropanol was added to dissolve the formazan crystals. Absorbance was measured at 570 nm using a microplate reader, and cell viability was calculated relative to the control group.

### 2.8. Antibacterial Performance Evaluation

Antibacterial activity was evaluated against *S. mutans* using a direct contact test. The tested groups were 0% MBG, 5% MBG, iRoot SP, and Vitapex. Freshly mixed materials were evenly spread onto the bottom of 6-well plates. A 1.6 mL suspension of *S. mutans* (5 × 10^7^ CFU/mL) was carefully added onto each material surface, and the plates were incubated anaerobically at 37 °C for 24 h. After incubation, bacterial suspensions were serially diluted tenfold, and aliquots from the appropriate dilution were plated onto agar plates. After a further 48 h of incubation under anaerobic conditions, colonies were photographed and counted.

### 2.9. Statistical Analysis

Data are presented as mean ± standard deviation (SD). Statistical analysis was performed using one-way analysis of variance (ANOVA) followed by Tukey’s post hoc test for multiple comparisons (Origin 2021). A value of *p* < 0.05 was considered statistically significant. Statistical significance was indicated as * *p* < 0.05 and ** *p* < 0.01, and non-significant differences were indicated as ns where appropriate.

## 3. Results and Discussion

### 3.1. Characterization and Practical Handling of the MBGN-Reinforced Sealer

**Figure 1:**
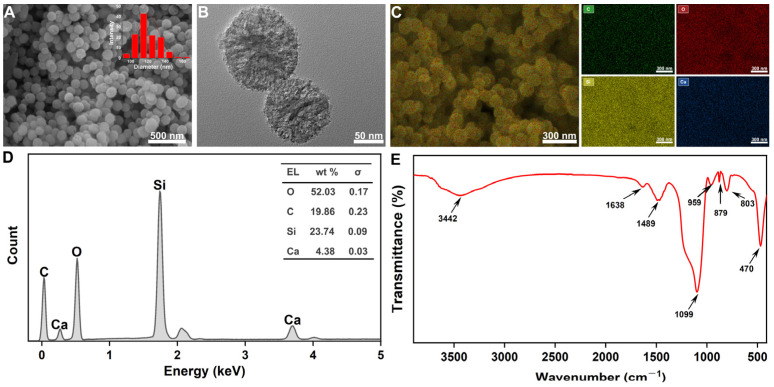
Morphological and physicochemical characterization of MBGN. (**A**) SEM image of MBGN with particle size distribution in the inset. (**B**) TEM image of MBGNs. (**C**) Elemental mapping of MBGN, including C, O, Si, and Ca. (**D**) EDS spectrum of MBGNs with elemental composition in the inset table. (**E**) FTIR spectrum of MBGN.

The MBGN-reinforced calcium silicate sealer was prepared by incorporating MBGN into a premixed calcium silicate-based matrix to provide a bioactive component for subsequent interfacial mineralization. SEM observation showed that the synthesized MBGN displayed a regular spherical morphology and homogeneous size distribution, with an average diameter of 120.0 ± 12.6 nm (Figure 1A). TEM imaging further showed well-defined internal mesoporous channels within the nanoparticles (Figure 1B), consistent with their mesoporous structure. This mesoporous architecture may facilitate fluid exchange and bioactive ion release, consistent with the mineralization-related function of MBGN [19,20]. EDS analysis further showed that the nanoparticles were mainly composed of Si and Ca (Figure 1C,D), consistent with the expected composition of mesoporous bioactive glass. The FTIR spectrum of MBGN (Figure 1E) showed characteristic absorption bands of a silicate-based bioactive glass structure. The broad band at approximately 3442 cm^−1^ and the peak at 1638 cm^−1^ were attributed to O–H stretching and H–O–H bending vibrations, respectively. The bands at approximately 1489 and 879 cm^−1^ were assigned to carbonate-related vibrations. The strong band at approximately 1099 cm^−1^ corresponded to Si–O–Si asymmetric stretching, while the bands at approximately 803 and 470 cm^−1^ were associated with Si–O–Si stretching and bending vibrations, respectively. The band at approximately 959 cm^−1^ was attributed to non-bridging oxygen-related Si–O groups [28,29]. These characteristic bands support the formation of silicate-network-based MBGN.

**Figure 2:**
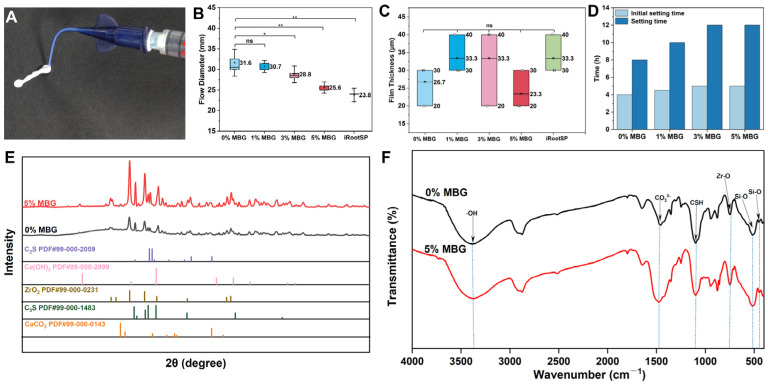
Handling-related properties and physicochemical characterization of the MBGN-reinforced calcium silicate sealer. (**A**) Gross appearance and syringe extrusion of the premixed sealer. (**B**) Flow diameter of different sealers. (**C**) Film thickness of different sealers. (**D**) Initial and final setting times of different MBG formulations (*n* = 3). (**E**) XRD patterns of the 0% MBG and 5% MBG formulations, with reference diffraction peaks of the main crystalline phases. (**F**) FTIR spectra of the 0% MBG and 5% MBG formulations. Data in (**B**,**C**) are presented as mean ± SD (*n* = 3). Setting times in (**D**) were measured with three specimens per group. ns, not significant; * *p* < 0.05; ** *p* < 0.01.

After confirming the structural characteristics of MBGN, we next examined whether their incorporation preserved the practical handling of the sealer system. As shown in Figure 2A, the prepared sealer exhibited a white and homogeneous paste-like appearance and could be smoothly extruded through the needle of a dental syringe, indicating suitable premixed injectability. Flowability testing further showed that all formulations remained within an acceptable handling range (Figure 2B). Among them, iRoot SP exhibited a relatively lower flow diameter of 23.8 ± 1.0 mm, whereas the 0% MBG group showed the highest value at 31.6 ± 1.93 mm. With increasing MBGN content, the flow diameter gradually decreased to 25.6 ± 0.8 mm in the 5% MBG group. Nevertheless, the MBGN-reinforced sealers still spread uniformly under compressive load, indicating that incorporation of MBGN did not impair practical operability. This moderate reduction in flowability may be related to nanoparticle-induced changes in paste rheology, while the formulations remained within a practical handling range [30,31]. Film thickness is also relevant to the ability of a sealer to establish intimate adaptation between gutta-percha and the canal wall. As shown in Figure 2C, no significant inter-group differences were observed between iRoot SP and the MBGN-reinforced formulations. These results indicate that incorporation of MBGN did not compromise the ability of the calcium silicate matrix to form a thin and continuous interfacial layer. Incorporation of MBGN also moderately prolonged the setting process (Figure 2D). The 0% MBG group reached initial and final setting at approximately 4 h and 8 h, respectively, whereas the 1% to 5% MBG groups exhibited initial and final setting times of approximately 5 h and 12 h. This effect may be associated with the mesoporous characteristics of MBGN, which could influence the early hardening behavior of the premixed calcium silicate system [18,32]. Nevertheless, all values remained well within the acceptable range specified by ISO 6876: 2012 (≤30 h) [27], indicating that the hybrid formulations retained a practically relevant setting window. XRD and FTIR analyses were further performed to characterize the physicochemical features of the 0% MBG and 5% MBG formulations. XRD patterns showed that both formulations contained the characteristic crystalline phases of the calcium silicate-based matrix, including tricalcium silicate, dicalcium silicate, zirconia, calcium carbonate, and hydration-related calcium hydroxide (Figure 2E), consistent with reported phase compositions of calcium silicate-based endodontic materials [32]. The similar diffraction profiles between the 0% MBG and 5% MBG groups indicate that MBGN incorporation did not substantially alter the primary crystalline constituents of the set sealer. FTIR spectra further revealed characteristic bands associated with hydroxyl groups, carbonate species, calcium silicate hydrate-related structures, Zr–O vibration, and Si–O–Si bonds (Figure 2F), in agreement with previously reported FTIR assignments for calcium silicate-based and cementitious materials [33,34]. These results suggest that the 5% MBG formulation retained the main physicochemical features of the calcium silicate-based matrix while incorporating a silicate-based bioactive component. Taken together, MBGN incorporation established a bioactive calcium silicate-based sealer system without compromising the practical handling characteristics required for root canal obturation. The hybrid formulations maintained suitable injectability, balanced flow behavior, stable film thickness, and acceptable setting kinetics, while providing a physicochemical basis for subsequent mineralization and sealing-related performance.

### 3.2. Dentin-Associated Mineralization Induced by the MBGN-Reinforced Sealer

**Figure 3:**
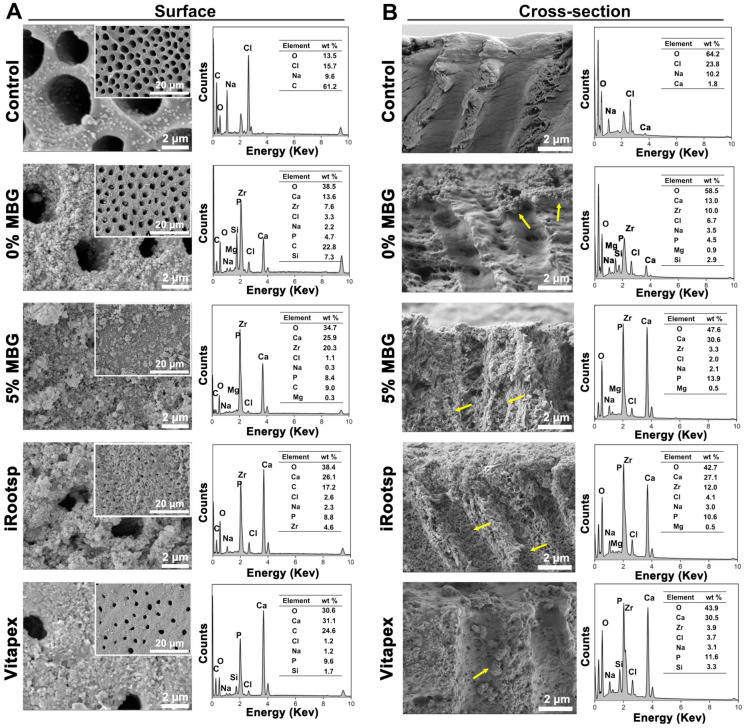
In vitro dentin-associated mineralization induced by different root canal sealers. (**A**) Surface SEM images and corresponding EDS spectra of dentin discs after 21 days of incubation with different sealers. Insets show low-magnification views of dentin surface coverage and dentinal tubule occlusion. (**B**) Cross-sectional SEM images and corresponding EDS spectra of the dentin-sealer interface and dentinal tubules. Yellow arrows indicate intratubular mineral deposits.

Dentin-associated mineralization is an important feature of bioactive root canal sealers because it is directly related to dentinal tubule occlusion and interfacial stabilization after obturation [35,36]. To examine whether incorporation of MBGN could enhance this behavior, dentin discs were immersed in SBF for 21 days in the presence of the experimental sealers and two clinically used reference materials, namely the calcium hydroxide-based sealer Vitapex and the calcium silicate-based sealer iRoot SP, while untreated dentin served as the control. As shown in Figure 3A,B, both surface and cross-sectional observations revealed clear differences in dentin-associated mineralization among the groups. The untreated control exhibited abundant open dentinal tubules and negligible mineral deposition, with no obvious Ca or P enrichment. Vitapex induced partial mineral deposition around tubular orifices, but the surface coverage and intratubular crystal formation were less continuous than those observed in the calcium silicate-based groups. In contrast, the 0% MBG, 5% MBG, and iRoot SP groups produced extensive surface mineral coverage accompanied by marked tubular occlusion. Cross-sectional SEM further showed that the 0% MBG group exhibited dense granular deposits at the tubule entrances and within the luminal space, whereas the 5% MBG and iRoot SP groups displayed abundant lamellar- and petal-like crystals lining the tubular walls. Representative EDS analysis further supported this mineralization trend. In the cross-sectional regions, the Ca content increased from 1.8 wt% in the untreated control to 13.0, 30.6, and 27.1 wt% in the 0% MBG, 5% MBG, and iRoot SP groups, respectively, accompanied by detectable P signals in the mineralized regions. These findings support the formation of calcium and phosphate-containing mineral deposits on dentin surfaces and within dentinal tubules. Collectively, the combined surface coverage, intratubular crystal deposition, increased Ca content, and detectable P signals indicate the formation of a denser dentin-associated mineral interface, which may support subsequent interfacial stabilization and improved sealing performance. This stronger mineralization behavior is likely related to the combined contribution of the calcium silicate matrix and the mesoporous bioactive glass phase. During immersion, MBGN may promote local ionic supersaturation through the release of calcium- and silicate-related species, while the calcium silicate phase provides a calcium-rich and alkaline microenvironment favorable for apatite-like precipitation [19,25,37]. These results suggest that MBGN may serve as a mineralization-promoting component within the calcium silicate matrix, thereby supporting dentin-associated mineral deposition and potential interfacial stabilization.

### 3.3. Apical Sealing Performance of the MBGN-Reinforced Sealer

**Figure 4:**
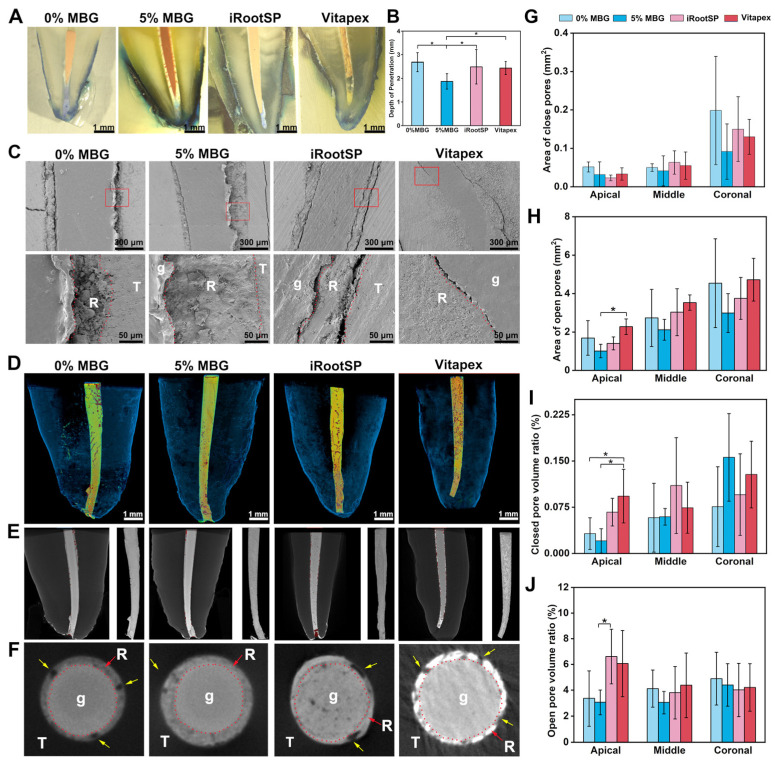
Apical sealing performance and microleakage evaluation of different root canal sealers. (**A**,**B**) Representative images and quantification of dye penetration. (**C**) Longitudinal SEM images of the tooth (T)–sealer (R)–gutta-percha (g) interfaces. Solid red box, magnified region; dashed red lines, sealer-tooth-gutta-percha interface. (**D**,**E**) Three-dimensional and longitudinal micro-CT reconstructions of obturated roots. (**F**) Representative transverse micro-CT images of the apical region. Red dashed lines indicate the gutta-percha boundary, and yellow arrows indicate interfacial voids or defects. (**G**–**J**) Quantitative porosity analysis in the apical, middle, and coronal thirds. Data in (**B**,**G**–**J**) are presented as mean ± SD (*n* = 6). * *p* < 0.05.

Apical microleakage is closely associated with endodontic treatment failure, because voids and interfacial discontinuities within the obturation system may serve as pathways for bacterial penetration and recolonization [4,38,39]. To assess whether MBGN incorporation improved sealing-related performance, dye penetration, SEM observation, and micro-CT analysis were performed after single-cone obturation. As shown in Figure 4A–C, both dye penetration and cross-sectional SEM observation revealed clear differences in apical sealing among the groups. The 0% MBG group exhibited the greatest dye penetration depth (2.68 ± 0.41 mm), whereas the 5% MBG group showed the lowest value (1.87 ± 0.32 mm), with a statistically significant difference between the two groups (*p* < 0.05). Vitapex and iRoot SP showed comparable penetration depths of 2.43 ± 0.27 mm and 2.48 ± 0.73 mm, respectively, both of which were greater than that of the 5% MBG group. SEM findings were consistent with these results. The 0% MBG group displayed a rougher microstructure with abundant pores and irregular crystalline deposits, indicative of a less compact interface. In contrast, the 5% MBG group exhibited a denser and more homogeneous structure with fewer visible pores and close adaptation to both dentinal walls and gutta-percha, without obvious interfacial gaps. Although iRoot SP showed a finer texture, discontinuities were still occasionally observed at the sealer–gutta-percha or sealer–dentin interfaces, while Vitapex also exhibited local defects and less compact adaptation. These results indicate that MBGN incorporation improved apical sealing not only by reducing dye leakage, but also by promoting formation of a more continuous interfacial structure.

Micro-CT analysis provided complementary three-dimensional evidence for internal porosity and sealing continuity (Figure 4D–J). In agreement with the dye penetration and SEM findings, the 0% MBG and Vitapex groups exhibited more pronounced void formation within the sealer matrix and less favorable apical coverage, whereas the 5% MBG group showed fewer and smaller pores together with more uniform apical filling. Quantitative pore analysis further confirmed this advantage. In the apical third, the open pore area was significantly lower in the 5% MBG group (1.01 ± 0.35 mm^2^) than in the Vitapex group (2.275 ± 0.40 mm^2^, *p* < 0.05), while both open and closed pore volumes were also lowest in the 5% MBG group. Because open pores are more directly associated with leakage pathways and closed pores reflect internal structural defects, the simultaneous reduction in both parameters supports the improved sealing quality of the 5% MBG formulation. Beyond the reduction in apical leakage, the improved sealer–dentin interfacial adaptation, reduced internal defects, and dentin-associated mineral deposition may collectively contribute to the enhanced sealing performance observed after MBGN incorporation. This effect may be related to the combination of initial adaptation supported by suitable flowability and subsequent bioactive interfacial densification mediated by calcium silicate hydration and MBGN-associated mineral deposition [19,40,41]. However, the present study did not directly simulate long-term aging or clinical failure conditions such as sealer dislodgement. Future studies should further evaluate long-term solubility, dimensional stability, ion release kinetics, and sealing durability under more clinically relevant conditions.

### 3.4. Cytocompatibility and Antibacterial Activity of the MBGN-Reinforced Sealer

**Figure 5:**
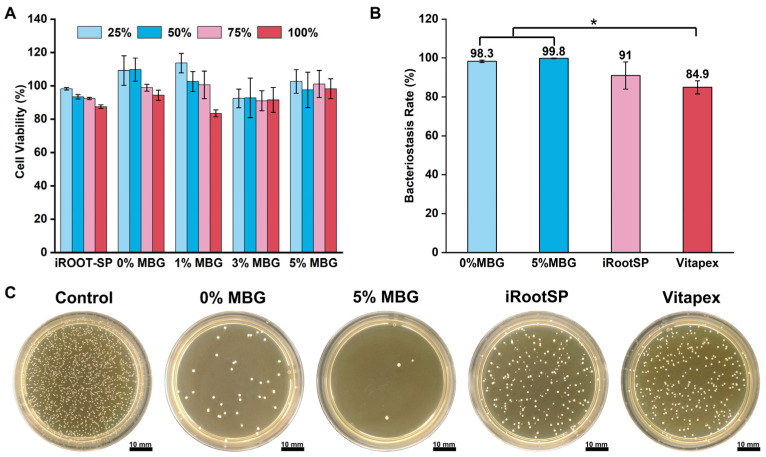
Cytocompatibility and antibacterial activity of different root canal sealers. (**A**) Viability of L929 fibroblasts cultured with sealer extracts at different concentrations. (**B**) Bacteriostasis rates against *S. mutans*. (**C**) Representative colony images of *S. mutans* after direct contact with different sealers. Data in (**A**) are presented as mean ± SD (*n* = 6), and data in (**B**) are presented as mean ± SD (*n* = 3). * *p* < 0.05.

The cytocompatibility of the cured MBGN-reinforced sealers was evaluated using the MTT assay. As shown in Figure 5A, cell viability remained above 80% across the tested extract concentrations, indicating an acceptable cytocompatibility profile. Notably, incorporation of MBGN did not adversely affect the cytocompatibility of the calcium silicate matrix. This may be related to the formation of a dense calcium silicate hydrate (C-S-H) network after hydration, which helps moderate excessive ion release and avoids abrupt alkalinity shifts [42,43]. The antibacterial activity of the sealers was then evaluated against *S. mutans*, a representative oral biofilm-forming bacterium [44]. As shown in Figure 5B,C, all calcium silicate-based sealers exhibited stronger antibacterial activity than the calcium hydroxide-based Vitapex group. Among them, the 5% MBG group achieved the highest bacteriostasis rate (99.8 ± 0.11%), which was significantly greater than that of Vitapex (*p* < 0.05) and slightly higher than that of iRoot SP. These results indicate that the calcium silicate-based matrix itself provided a baseline antibacterial effect, while incorporation of MBGN further strengthened this activity. This enhancement may be related to hydration-derived alkalinity and ionic modulation within the local microenvironment [45,46]. In the present system, MBGN may contribute by providing a mesoporous bioactive phase capable of ion exchange and release of calcium- and silicate-related species [47]. The enhanced mineralization tendency may also support antibacterial performance by reducing favorable sites for bacterial attachment [48,49,50]. Nevertheless, the antibacterial evaluation in this study was limited to *S. mutans*, and the proposed role of alkalinity and ion release was mainly inferred from material composition and previous studies rather than directly verified by pH or ion-release measurements. Future studies should include E. faecalis and polymicrobial endodontic biofilm models, together with pH evolution, ion release profiling, and long-term antibacterial assessments, to further clarify the antibacterial mechanism and clinical relevance of the MBGN-reinforced sealer.

## 4. Conclusions

In this study, an injectable MBGN-reinforced calcium silicate root canal sealer was developed as a bioactive strategy to improve the functional performance of endodontic obturation materials. The incorporation of MBGN preserved practical handling characteristics while improving dentin-associated mineralization, apical sealing performance, and antibacterial activity against *S. mutans*. Among the tested formulations, the 5% MBG formulation showed the most favorable overall performance, as reflected by reduced microleakage, lower porosity, enhanced interfacial mineral deposition, and strong antibacterial activity. These findings suggest that MBGN incorporation is a feasible approach for improving the sealing-related and biological performance of calcium silicate-based root canal sealers. Nevertheless, this study was mainly based on in vitro and ex vivo evaluations. Further long-term and in vivo studies are needed to confirm the durability, biological response, and translational potential of this sealer under more clinically relevant conditions.

## Figures and Tables

**Table 1:** Powder compositions of the MBGN sealers.

	ZrO_2_	C_3_S	C_2_S	CaCO_3_	MBGNs
0% MBG	40%	30%	18%	12%	0%
1% MBG	40%	29.5%	17.7%	11.8%	1%
3% MBG	40%	28.5%	17.1%	11.4%	3%
5% MBG	40%	27.5%	16.5%	11%	5%

**Table 2:** Compositions of the tested sealer materials.

Materials	Powder Composition	Liquid Carrier
MBGN sealer	ZrO_2_, C_3_S, C_2_S, CaCO_3_, MBGN	PEG200, Tween-80
iRoot SP [24]	ZrO_2_, calcium silicate, calcium phosphate, calcium hydroxide, filler	Thickening agent
Vitapex [26]	Calcium hydroxide, sodium carboxymethyl cellulose, iodoform, polymethylsiloxane	Olive oil

## Data Availability

The original contributions presented in this study are included in the article; further inquiries can be directed to the corresponding author.

## Abbreviations

The following abbreviations are used in this manuscript:

CFU	colony-forming unit
C_2_S	dicalcium silicate
C_3_S	tricalcium silicate
CTAB	cetyltrimethylammonium bromide
MBG	mesoporous bioactive glass
MBGN	mesoporous bioactive glass nanoparticles
MTA	mineral trioxide aggregate

## References

[1] Tiburcio-Machado C.S., Michelon C., Zanatta F.B., Gomes M.S., Marin J.A., Bier C.A. (2021). The global prevalence of apical periodontitis: A systematic review and meta-analysis. Int. Endod. J..

[2] Duncan H.F., Kirkevang L.L., Peters O.A., El-Karim I., Krastl G., Del Fabbro M., Chong B.S., Galler K.M., Segura-Egea J.J., Kebschull M. (2023). Treatment of pulpal and apical disease: The European Society of Endodontology (ESE) S3-level clinical practice guideline. Int. Endod. J..

[3] Gulabivala K., Ng Y.L. (2023). Factors that affect the outcomes of root canal treatment and retreatment—A reframing of the principles. Int. Endod. J..

[4] Snigdha N.T., Karobari M.I. (2025). Bacterial Leakage Testing in Dentistry: A Comprehensive Review on Methods, Models, and Clinical Relevance. Scientifica.

[5] Gillen B.M., Looney S.W., Gu L.S., Loushine B.A., Weller R.N., Loushine R.J., Pashley D.H., Tay F.R. (2011). Impact of the quality of coronal restoration versus the quality of root canal fillings on success of root canal treatment: A systematic review and meta-analysis. J. Endod..

[6] Wang Z. (2015). Bioceramic materials in endodontics. Endod. Top..

[7] Alvarez-Vasquez J.L., Erazo-Guijarro M.J., Dominguez-Ordonez G.S., Ortiz-Garay E.M. (2024). Epoxy resin-based root canal sealers: An integrative literature review. Dent. Med. Probl..

[8] Sfeir G., Zogheib C., Patel S., Giraud T., Nagendrababu V., Bukiet F. (2021). Calcium Silicate-Based Root Canal Sealers: A Narrative Review and Clinical Perspectives. Materials.

[9] Mohammadi Z., Dummer P.M. (2011). Properties and applications of calcium hydroxide in endodontics and dental traumatology. Int. Endod. J..

[10] Estrela C., Bammann L.L., Pimenta F.C., Pecora J.D. (2001). Control of microorganisms in vitro by calcium hydroxide pastes. Int. Endod. J..

[11] ØRstavik D.A.G. (2006). Materials used for root canal obturation: Technical, biological and clinical testing. Endod. Top..

[12] Kokkas A.B., Boutsioukis A., Vassiliadis L.P., Stavrianos C.K. (2004). The influence of the smear layer on dentinal tubule penetration depth by three different root canal sealers: An in vitro study. J. Endod..

[13] Camilleri J., Mallia B. (2011). Evaluation of the dimensional changes of mineral trioxide aggregate sealer. Int. Endod. J..

[14] Torabinejad M., Parirokh M., Dummer P.M.H. (2018). Mineral trioxide aggregate and other bioactive endodontic cements: An updated overview—Part II: Other clinical applications and complications. Int. Endod. J..

[15] Donnermeyer D., Burklein S., Dammaschke T., Schafer E. (2019). Endodontic sealers based on calcium silicates: A systematic review. Odontology.

[16] Skallevold H.E., Rokaya D., Khurshid Z., Zafar M.S. (2019). Bioactive Glass Applications in Dentistry. Int. J. Mol. Sci..

[17] Dai L.L., Mei M.L., Chu C.H., Lo E.C.M. (2019). Mechanisms of Bioactive Glass on Caries Management: A Review. Materials.

[18] Jung M.K., Park S.C., Kim Y.J., Park J.T., Knowles J.C., Park J.H., Dashnyam K., Jun S.K., Lee H.H., Lee J.H. (2022). Premixed Calcium Silicate-Based Root Canal Sealer Reinforced with Bioactive Glass Nanoparticles to Improve Biological Properties. Pharmaceutics.

[19] Zheng K., Sui B., Ilyas K., Boccaccini A.R. (2021). Porous bioactive glass micro- and nanospheres with controlled morphology: Developments, properties and emerging biomedical applications. Mater. Horiz..

[20] Zhu Y., Zhang X., Chang G., Deng S., Chan H.F. (2025). Bioactive Glass in Tissue Regeneration: Unveiling Recent Advances in Regenerative Strategies and Applications. Adv. Mater..

[21] Shearer A., Montazerian M., Sly J.J., Hill R.G., Mauro J.C. (2023). Trends and perspectives on the commercialization of bioactive glasses. Acta Biomater..

[22] Mobeen B., Muhammad N., Choudhury M., Feroz A., Feroz S. (2026). Mesoporous Bioactive Glass: Preparation, Characterisation, and Emerging Applications in Regenerative Medicine and Dentistry. Int. Dent. J..

[23] Simila H.O., Anselmi C., Cardoso L.M., Dal-Fabbro R., Beltran A.M., Bottino M.C., Boccaccini A.R. (2024). Sol-gel-derived calcium silicate cement incorporating collagen and mesoporous bioglass nanoparticles for dental pulp therapy. Dent. Mater..

[24] Lin G.S.S., Sim D.H.H., Luddin N., Lai J.C.H., Ghani H.A., Noorani T.Y. (2023). Fabrication and characterisation of novel algin incorporated bioactive-glass 58S calcium-silicate-based root canal sealer. J. Dent. Sci..

[25] Sui B., Xu Z., Xue Z., Xiang Y., Zhou T., Beltran A.M., Zheng K., Liu X., Boccaccini A.R. (2023). Mussel-Inspired Polydopamine Composite Mesoporous Bioactive Glass Nanoparticles: An Exploration of Potential Metal-Ion Loading Platform and In Vitro Bioactivity. ACS Appl. Mater. Interfaces.

[26] de Souza L.C., Neves G.S.T., Kirkpatrick T., Letra A., Silva R. (2023). Physicochemical and biological properties of AH plus bioceramic. J. Endod..

[27] (2012). Dentistry—Endodontic Sealing Materials.

[28] Vaid C., Murugavel S. (2013). Alkali oxide containing mesoporous bioactive glasses: Synthesis, characterization and in vitro bioactivity. Mater. Sci. Eng. C Mater. Biol. Appl..

[29] Zheng K., Kang J., Rutkowski B., Gaweda M., Zhang J., Wang Y., Founier N., Sitarz M., Taccardi N., Boccaccini A.R. (2019). Toward Highly Dispersed Mesoporous Bioactive Glass Nanoparticles with High Cu Concentration Using Cu/Ascorbic Acid Complex as Precursor. Front. Chem..

[30] Sharma A., Gupta S., Noman Husain M., Chaudhary S. (2025). Factors affecting the rheology of cement-based composites: A review. J. Am. Ceram. Soc..

[31] Yun N., Kim H.J., Kwon J., Kim S.Y., Kim D.S. (2025). Enhancing dental sealant performance: Effects of mesoporous bioactive glass and 10-MDP on adhesion and remineralization. J. Dent..

[32] Rezvani M., Düzgüneş N., Kesharwani P. (2026). Multifaceted application of bioactive glass nanoparticles in dentistry: The state of the art. Applications of Nanomaterials in Dentistry.

[33] Yusuf M.O. (2023). Bond Characterization in Cementitious Material Binders Using Fourier-Transform Infrared Spectroscopy. Appl. Sci..

[34] Timón V., Torrens-Martin D., Fernández-Carrasco L.J., Martínez-Ramírez S. (2023). Infrared and Raman vibrational modelling of β-C2S and C3S compounds. Cem. Concr. Res..

[35] Kaushik S.N., Kim B., Walma A.M., Choi S.C., Wu H., Mao J.J., Jun H.W., Cheon K. (2016). Biomimetic microenvironments for regenerative endodontics. Biomater. Res..

[36] Baras B.H., Melo M.A.S., Thumbigere-Math V., Tay F.R., Fouad A.F., Oates T.W., Weir M.D., Cheng L., Xu H.H.K. (2020). Novel Bioactive and Therapeutic Root Canal Sealers with Antibacterial and Remineralization Properties. Materials.

[37] Nawaz Q., Varlik E., Mutlu N., Michálek M., Boccaccini A.R.J.M.C. (2025). Germanium-containing mesoporous bioactive glass nanoparticles (MBGNs) as a new member of the MBGNs family: Synthesis and preliminary characterization. MRS Commun..

[38] Muliyar S., Shameem K.A., Thankachan R.P., Francis P.G., Jayapalan C.S., Hafiz K.A. (2014). Microleakage in endodontics. J. Int. Oral Health.

[39] Badawi N.M., Kataia M.M., Mousa H.A., Afshari M. (2025). Advancements in Root Canal Therapy: Translational Innovations and the Role of Nanoparticles in Endodontic Treatment. J. Nanotechnol..

[40] Wang Y., Yu J., Zhang X., Tang C., Wang K., Zhao Y., Huang C., Yang H. (2026). Recent Advances of Mesoporous Nanoparticles in the Management of Dentin Hypersensitivity. Adv. Funct. Mater..

[41] Migneco C., Fiume E., Verne E., Baino F. (2020). A Guided Walk through the World of Mesoporous Bioactive Glasses (MBGs): Fundamentals, Processing, and Applications. Nanomaterials.

[42] Hamdy T.M., Galal M.M., Ismail A.G., Saber S. (2024). Physicochemical properties of AH plus bioceramic sealer, Bio-C Sealer, and ADseal root canal sealer. Head Face Med..

[43] Nicoleau L., Nonat A. (2016). A new view on the kinetics of tricalcium silicate hydration. Cem. Concr. Res..

[44] Guo X., Wang X., Shi J., Ren J., Zeng J., Li J., Li Y. (2024). A review and new perspective on oral bacteriophages: Manifestations in the ecology of oral diseases. J. Oral Microbiol..

[45] Komabayashi T., Colmenar D., Cvach N., Bhat A., Primus C., Imai Y. (2020). Comprehensive review of current endodontic sealers. Dent. Mater. J..

[46] Iqbal K., Alhomrany R., Berman L.H., Chogle S. (2023). Enhancement of Antimicrobial Effect of Endodontic Sealers Using Nanoparticles: A Systematic Review. J. Endod..

[47] Wu C., Chang J. (2014). Multifunctional mesoporous bioactive glasses for effective delivery of therapeutic ions and drug/growth factors. J. Control. Release.

[48] He J., Yang J., Li M., Li Y., Pang Y., Deng J., Zhang X., Liu W. (2022). Polyzwitterion Manipulates Remineralization and Antibiofilm Functions against Dental Demineralization. Acs Nano.

[49] Kaya S., Cresswell M., Boccaccini A.R. (2018). Mesoporous silica-based bioactive glasses for antibiotic-free antibacterial applications. Mater. Sci. Eng. C Mater. Biol. Appl..

[50] Li Q., Liu J., Liu H., Sun Y., Xu Y., Wang K., Huang W., Liao L., Wang X. (2023). Multifunctional magnesium organic framework-based photothermal and pH dual-responsive mouthguard for caries prevention and tooth self-healing promotion. Bioact. Mater..

